# A Retrospective Five-Year Study of Cardiovascular Risk Assessment and Risk-Based Interventions Among Hypertensive Patients in Nairobi Hospital, Kenya

**DOI:** 10.7759/cureus.46097

**Published:** 2023-09-27

**Authors:** Kevin O Ndede, Zahid Khan, Florence K Akumiah, Martin Wanyoike

**Affiliations:** 1 Internal Medicine/Medical Physiology, Kenya Methodist University, Nairobi, KEN; 2 Acute Medicine, Mid and South Essex NHS Foundation Trust, Southend-on-Sea, GBR; 3 Cardiology, Bart’s Heart Centre, London, GBR; 4 Cardiology and General Medicine, Barking, Havering and Redbridge University Hospitals NHS Trust, London, GBR; 5 Cardiology, Royal Free Hospital, London, GBR; 6 Internal Medicine, Korle-Bu Teaching Hospital, Accra, GHA; 7 Cardiology, The Nairobi Hospital, Nairobi, KEN

**Keywords:** categorical variables, hypertensive individuals on statins, national ambulatory medical care survey, therapeutic lifestyle interventions, hypertensive patients, cvd risk assessment, cardiovascular disease prevention, cardiovascular disease risk, cardiovascular disease (cvd), retrospective research

## Abstract

Introduction

Cardiovascular disease (CVD) is a leading cause of global morbidity and mortality. It is projected that the prevalence of CVD will continue to rise in developing countries, largely driven by an increase in the prevalence of potentially modifiable risk factors. Atherosclerotic cardiovascular risk assessment among individuals with risk factors for CVD but without CVD is an inexpensive and viable strategy in CVD risk stratification and prevention. Despite the known benefits of CVD risk assessment, it is not well established whether physicians/ cardiologists in Kenya comply with the guideline-recommended practice of CVD risk stratification as a prerequisite for initiation of primary CVD preventive interventions.

Aims and objectives

This study was designed to audit the utilization of cardiovascular risk assessment tools in risk stratification of hypertensive individuals and physician provision of risk-based primary CVD prevention interventions.

Results

A five-year (2017-2022) retrospective study of patients’ medical records was conducted in December 2022 at the PrimeCare cardiology clinic in Nairobi Hospital, Kenya. Data were collected from 373 patients' medical records retrospectively. The data were analyzed using IBM SPSS Statistics for Windows, Version 25 (Released 2017; IBM Corp., Armonk, New York, United States). The mean age of the patients was 60 years with the majority being female (54%). The mean BMI was 30.3 kg/m^2^ while the mean systolic and diastolic pressure was 140mmHg and 80mmHg, respectively. Only 2.1% of participants were current smokers. The national or alternative guideline-recommended CVD risk assessment tool was used in 0.3% and 2.4%, respectively. The 10-year CVD risk score was documented in only 1.3%. The majority of the participants (93%) had low CVD risk. Half of the patients were taking statins for primary prevention while > 60% of them had been offered therapeutic lifestyle advice.

Conclusion

The study revealed poor compliance with guideline-recommended CVD risk assessment tools and documentation of the CVD risk level. However, there was above-average adherence to documentation of therapeutic lifestyle measures for primary CVD prevention.

## Introduction

The global burden of cardiovascular disease and risk factor report depicts a near doubling of the prevalence of cardiovascular disease and a 50% increase in cardiovascular disease (CVD) mortality between 1990 and 2019 [1]. The current high prevalence and projected increase in CVD prevalence will most likely be driven by the increase in most of the potentially modifiable cardiovascular disease risk factors: unhealthy diets, hypertension, diabetes, overweight, obesity, and cigarette smoking. Prevention of the growing burden of CVD requires appropriate measures to reduce these modifiable risk factors [2]. In Kenya, a high burden of cardiovascular risk factors was demonstrated by the WHO STEPs survey which revealed that the majority of the adult population (75.6%) had four to six risk factors for CVD while 10% had seven or more CVD risk factors [3]. Despite the high prevalence of risk factors, an initial audit in Kenya among general practitioners at a public hospital in 2013 found zero uptake of CVD risk assessment with a follow-up audit among the same cohort of health workers five months later revealing no improvement in uptake of CVD risk assessment despite strategies to encourage its use [4].

Atherosclerotic cardiovascular risk assessment among individuals with risk factors for CVD but without cardiovascular disease is an inexpensive and viable strategy in CVD risk stratification and prevention. CVD risk stratification helps in the prioritization of individuals for further formal and more elaborate risk assessment, initiation of risk-based interventions, monitoring of temporal variation in an individual’s risk profile, prioritization of treatment resources, and mitigation of further risk factor accumulation [3,5]. Internal Medicine physicians and cardiologists in Kenya are often at the apex of care of individuals with increased cardiovascular risk but it is not well known to what extent these clinicians adhere to Kenya’s national CVD service delivery model that recommends cardiovascular risk screening, assessment, and management of various categories of individuals [6]. We designed a study to audit the practice of CVD risk stratification and risk-based interventions among hypertensive patients managed by physician-internists and cardiologists at a cardiology practice in Nairobi, Kenya.

This study aimed to evaluate the utilization of cardiovascular risk assessment tools in the risk stratification of hypertensive individuals and to evaluate whether primary CVD prevention interventions by clinicians are based on cardiovascular risk stratification.

## Materials and methods

**Figure 1:**
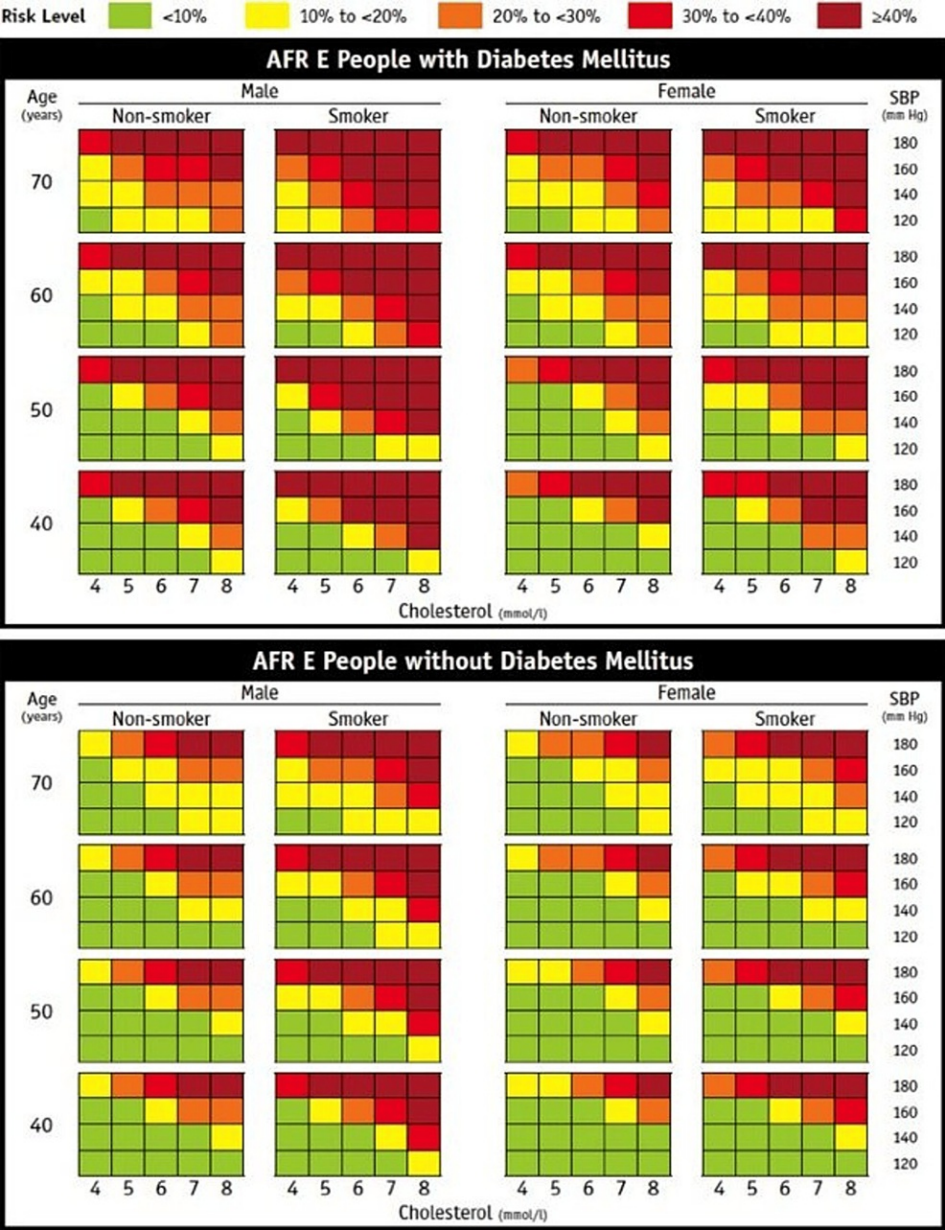
WHO CVD risk laboratory-based charts for Eastern Sub-Saharan Africa Permission obtained from authors to reproduce the figure [7]. WHO: World Health Organization, CVD: cardiovascular disease

The recommended tool for risk assessment is the World Health Organization/ International Society of Hypertension (WHO/ISH) East African region prediction charts (Figure 1). The charts that can be used are of two types: laboratory and non-laboratory. The non-laboratory charts estimate cardiovascular risk without incorporating cholesterol measurements. They are therefore more useful where the cost of cholesterol measurement is an impediment or access to a laboratory is limited. The other risk factors included in the risk assessment are common to both types of charts and include systolic blood pressure, age, sex, diabetes status, and smoking status.

The study was conducted as a retrospective study of the medical records of hypertensive individuals who attended the clinic over the last five years between 2017 and 2022. A modified WHO/ISH data abstraction tool was used to extract relevant data from 373 eligible medical records between the dates of December 2022 and January 2023. The following criteria formed the core basis of the study:

Was the Kenyan national guideline-recommended World Health Organization/ International Society of Hypertension (WHO/ISH) cardiovascular risk assessment tool for the East African region used over the last year? The eligible medical records were reviewed for the risk score documentation in the preceding year of the patient's clinic visit. The dichotomized response was a yes or no. 

Was an alternative CVD risk assessment tool used instead of the WHO/ISH? In the absence of documented use of the charts, we checked through the medical records to establish the use of any other guideline-recommended risk assessment tool. The finding was recorded as yes or no.

Was a 10-year atherosclerotic cardiovascular disease (ASCVD) risk estimate recorded in the file over the last year? We looked through the medical records to identify documentation of an aggregate 10-year CVD risk score. The finding was recorded as yes or no.

What would the calculated WHO/ISH risk score be based on the medical record data? We utilized data available in the medical records (age, sex, systolic blood pressure, total cholesterol measurement, smoking status, and diabetes status) to calculate the CVD risk score using the WHO/ISH risk calculator. The calculated risk score was recorded as a percentage and categorized into risk levels (< 5%, 5% - <10%, 10% - < 20%, 20% - >30%, ≥ 30%).

Did the doctor prescribe a statin? We recorded a yes response if a statin was prescribed in the medical records and a no if there wasn't.

Was the prescription of the statin necessary? This was determined for individuals whose medical records had a statin prescription. The necessity was a function of the calculated East African region (AFR E) WHO/ISH score in the national guideline recommendation for statin therapy among individuals with a risk above 20%. For individuals who had risk levels below 20% but with statin prescriptions in their medical records, the prescription was deemed unmerited and recorded in our data as such.

Were the following primary prevention strategies offered to the patient and documented in the medical record? We perused the medical records to establish whether specific advice on the following healthy lifestyle measures was issued: Healthy diet: the specific advice evaluated included a low salt diet, adequate portions of fruits and vegetables, and low dietary fat and cholesterol; smoking cessation; weight management, and physical exercise.

Sample size

The sample size was determined using Table 1 recommended by the University of Bristol Hospital NHS Trust foundation guide for clinical audits and retrospective studies [8]. The target for meeting this standard is set at 70% with a 5% margin of inaccuracy. This would mean that based on a 95% confidence interval, the true value would lie between 65% and 75%.

**Table 1:** Sample size calculation based on (How to Guides | University Hospitals Bristol NHS Foundation Trust, 2005) Source: [7]

Population size	Sample size: 95% confidence; +/- 5%
50	44
100	79
150	108
200	132
500	217
1000	278
2000	322
5000	357

Table 1 relies on the assumption of a 50% expected incidence rate of the outcome of interest and a 50% compliance rate to the standards to estimate the sample size required to achieve a 95% confidence interval and 5% precision.

Data analysis

Statistical analysis in this study was performed using IBM SPSS Statistics for Windows, Version 25 (Released 2017; IBM Corp., Armonk, New York, United States). Descriptive statistics including frequency tables, proportions, mean, and standard deviation were used to describe the data. Cross tabulations were used to provide further insights between risk assessments and risk-based interventions.

## Results

Patient demographics

Most study participants were female 202 (54.16%) and consisted of middle-aged individuals with a mean age of 60 years (SD 12.7), ranging from 38 to 95 years. The mean BMI was 30.3 kg/m^2^ (SD 6.14) while the mean systolic blood pressure and mean diastolic blood pressure were 139.9 mmHg and 81.1 mmHg (SD- 20.3 and 12.3) respectively. Most study participants 345 (92.5%) were never smokers, 20 (5.4 %) were former smokers, and 8 (2.1%) were current smokers. We noted that 80 (21.4%) study participants had diabetes documented in their medical records in this study (Table 2). The mean age of the individuals studied was between 45 and 69 years. The mean BMI of 30.3 kg/m^2^ suggests that the majority of the hypertensive individuals studied were obese. Obesity confers a two-fold increase in the odds of hypertension in the African population. The studied population had a lower prevalence of current smokers 2.1%. Our study showed a low documentation of advice on smoking cessation.

**Table 2:** Characteristics of study participants BMI: Body mass index; BP: blood pressure

Characteristics (n= 373)	Mean	Standard deviation
Age in years	60.0	12.8
Weight (Kg)	83.1	16.8
Height (cm)	165.8	9.12
BMI (Kg/m^2^)	30.3	6.13
Systolic BP (mmHg)	140.0	20.3
Diastolic BP (mmHg)	81.1	12.3
Categorical variables	Type	Percentage
Sex	Female	202 (54.2%)
	Male	171 (45.8%)
Smoking status	Current	8 (2.1%)
	Former	20 (5.4%)
	Never	345 (92.5%)

Findings on CVD risk assessment

In the majority of the medical records, 372 of 373 (99.7%) did not have any documented use of the Kenya national guideline cardiovascular disease risk assessment tool (AFR E tool/chart), while only 9 (2.4%) had documented use of an alternative guideline-recommended cardiovascular disease risk assessment tool (Table 3). Additionally, 368 records (98.7%) lacked a documented CVD risk score within the last 12 months (January 2022 - January 2023). An evaluation of the association between categorical variables and risk classification of CVD revealed a significant association for sex ≤ 4.298 p = 0.038) but not for smoking ≤ 0.76 p = 0.684) nor diabetes ≤ 2.876 p = 0.133). When assessed using the t-test, age (p=0.152), BMI (p=0.901), systolic blood pressure (p=0.446), and diastolic blood pressure (p=0.730) were not significantly associated with the risk classification of CVD.

**Table 3:** Compliance with cardiovascular disease risk assessment CVD: Cardiovascular disease

Characteristic under assessment	Positive n (%)	Negative n (%)
Use of national guideline CVD risk assessment tool.	1 (0.3)	372 (99.7)
Use of alternative guideline CVD risk assessment tool.	9 (2.4)	363 (97.6)
Documented CVD risk score.	5 (1.3)	368 (98.7)

In-study CVD risk assessment and classification

The study calculated the participants’ CVD risk based on the recommended AFR E CVD risk assessment tool. In most of the hypertensive patients, 347 (93%) under study were classified as low cardiovascular disease risk (CVD risk < 20%) while 26 (7.0%) were classified as high cardiovascular disease risk patients (CVD risk ≥ 20%) as shown in Figure 1. Of the 26 hypertensive individuals (7%) who were categorized as high CVD risk, 17 (65.4%) were male while 9 (34.6%) were female. Among the study participants with diabetes, the majority 71 (88.7%) were categorized as low CVD risk. The presence of the diagnosis of diabetes did not show an association with the risk assessment for CVD.

**Figure 2:**
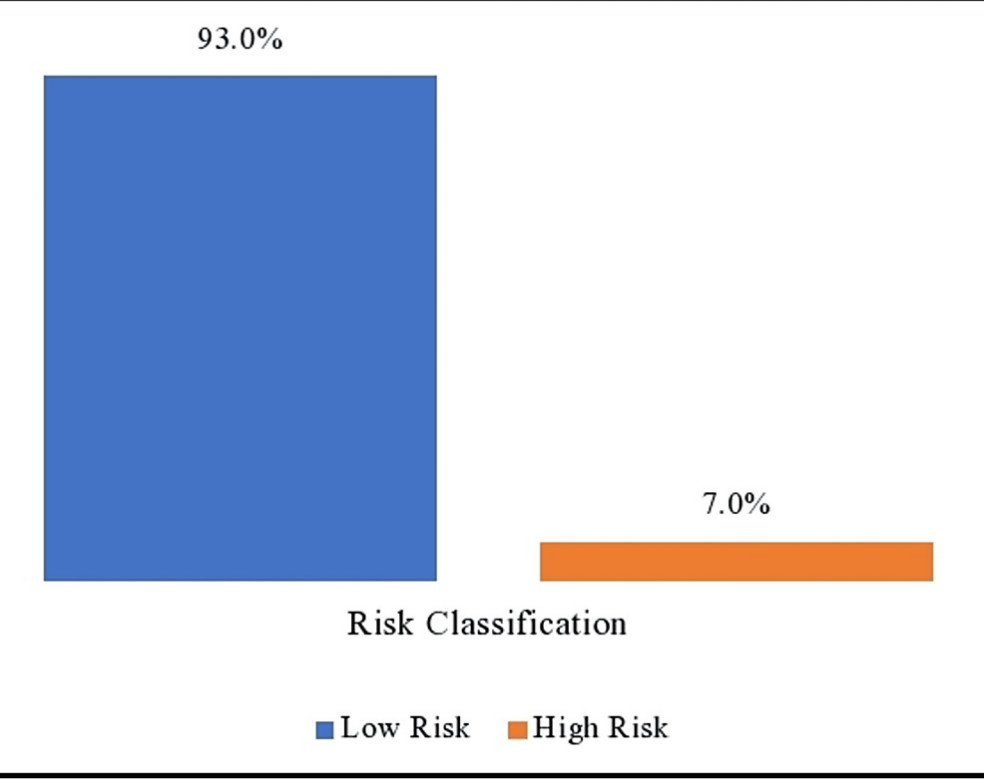
Stratification of cardiovascular disease risk assessment

Primary interventions

During the period under audit, results show that about half of the hypertensive patients, 184 (49.1%) were on a statin prescription. The majority of these patients on statins, 164 (44%), were classified as low risk for CVD while the remaining 19 (5.1%) were categorized as high risk (Table 4). Hypertensive individuals on statins were more likely to be in the high CVD risk category. Only seven (1.9%) of the hypertensive patients who were classified as high CVD risk were not on pharmacological intervention. The results of risk-based utilization of pharmacological intervention are shown in Table 4. Of the 80 diabetic individuals, 55 (68%) individuals had documented statin prescriptions while 25 (32%) individuals on pharmacological management of diabetes did not have a documented statin prescription.

**Table 4:** Proportions of hypertensive patients on statins stratified by risk category

Total number of patients		Risk Classification		Total
		Low Risk	High Risk	
On Statin	No	183 (49.1%)	(7) 1.9%	50.9%
	Yes	164 (44.0%)	(19) 5.1%	49.1%
Total		347 (93.0%)	(26)7.0%	100.0%

Therapeutic lifestyle interventions

The documentation of therapeutic lifestyle interventions for CVD risk reduction found that 258 (69.2%) of individuals had received advice on weight management and physical activity, 259 (69.4%) had received advice on dietary salt reduction, adequate portions of fruits and vegetables, and low cholesterol diet while 366 (98%) of current smokers did not have documented advice to stop smoking. The CVD risk category did not significantly influence the provision of any of the therapeutic lifestyle interventions listed in Table 5. The student t-test value for various categories is provided in Table 5.

**Table 5:** Summary of the proportion of patients who received advice on non-pharmacological interventions

Intervention	Outcome	Risk classification		Total	Statistical t-test
		Low risk	High risk		
Weight management	Yes (%)	64.1	5.1	69.2	t= 2.6991 p=0.2259
	No (%)	29.0	1.9	30.8	
The guideline recommends physical activity	Yes (%)	64.1	5.1	69.2	t= 2.6991 p=0.2259
	No (%)	29.0	1.9	30.8	
Advice on smoking cessation (Current smokers)	Yes (%)	2.1	0.0	2.1	t=1.0513 p=0.4841
	No (%)	90.9	7.0	97.9	
Dietary salt reduction	Yes (%)	64.3	5.1	69.4	t=2.6543 p=0.2294
	No (%)	28.7	1.9	30.6	

## Discussion

This study depicts that compliance with both national and alternative guideline-recommended CVD risk assessment tools is poor among physicians and cardiologists with a bias against the national tool. The mean age of the individuals studied was congruent with the Kenya population data which showed that the majority of hypertensive individuals in Kenya are aged between 45 and 69 years [9]. Studies among physicians from different parts of Africa indicate that despite high levels of awareness of global CVD risk assessment tools and the benefits of CVD risk assessment, only a minority of physician practitioners between 23% and 28% use the tools [10,11]. Similar to our study, Egyptian physicians’ reliance on alternative guideline-recommended global CVD risk assessment tools was higher than the use of their national guideline-recommended tool [8]. The mean BMI of hypertensive patients was 30.3 kg/m^2^ suggesting that this is higher than the Kenya national population average where only 9% of adults are classified as obese.

Our study demonstrated that documentation of 10-year global CVD risk score was poor (1.3%) despite the presence of documented individual CVD risk factors, yet this was consistent with findings from other African studies whereby in > 80% of the cases physicians documented individual CVD risk factors but did not compute global CVD risk subsequently. Although our study did not assess any determinants of the poor usage of cardiovascular risk assessment tools nor documentation of global CVD risk score, these are likely to be similar to findings from other studies from Egypt and Nigeria which reported several barriers to the use of CVD risk assessment tools by physicians such as time constraints in the clinic, cost of investigations, poor familiarity with the use of the tools and patient fears of knowing their risk status [10,11].

The majority of the participants studied (93%) had low cardiovascular disease risk (AFR E global CVD risk score < 20%) while males were two times more likely to be high-risk individuals than females. We observed that the majority of individuals with a low CVD risk score (90%) were on statin therapy contrary to the Kenya national guideline recommendation on primary pharmacological prevention therapy with a statin [6]. The international guideline recommended practice to initiate statin therapy among diabetic patients for primary CVD prevention irrespective of the cholesterol measurement or the global CVD risk score is a potential reason for the disproportionate statin prescription relative to total CVD risk score [12]. Our data show that 21.4% of individuals under study were on pharmacological treatment for diabetes mellitus. The majority of individuals on pharmacological management of diabetes also had documented statin use, However, one-third of diabetic individuals were not on statin therapy representing a missed opportunity for primary CVD prevention consistent with a study in Botswana which found that the majority (54.5%) of individuals with diabetes were not on a statin medication despite being eligible [13].

Concerning non-pharmacological primary prevention strategies, the study revealed that except for advice on smoking cessation, which was low, the majority of participants (about 70%) had documented advice on therapeutic lifestyle interventions despite being in the low CVD risk category. Our study population had a smoking prevalence of 2.1% compared to the national average of 10% which could be due to patients' awareness of the combined higher cardiovascular risk of hypertension and smoking. Our physicians from the concerned clinic offered healthy lifestyle advice to a higher proportion of individuals in comparison to healthy lifestyle advice offered by health workers during a Kenyan general population stepwise survey [14]. Table 6 provides a comparison of healthy lifestyle advice between our audit and the Kenyan national survey. Despite the seemingly good performance in offering healthy lifestyle measures, the service can still be improved since about one-third of patients who attended the clinic were not offered advice on healthy lifestyle measures.

**Table 6:** Comparison of the proportion of individuals who received healthy lifestyle advice between the present study and a previous national survey Source: [12]

Lifestyle advice	% Receiving advice in the present audit	% Receiving advice -STEPwise survey
Maintain healthy body weight or lose weight	69.2	11.4
Physical activity	69.2	11.9
Reduction in dietary salt	69.4	10.3
Smoking cessation	2.1	8.3
Adequate portions of fruits and vegetables	69.4	20.5
Reducing dietary cholesterol	69.4	11.4

The WHO data show that 86% of deaths due to non-communicable diseases in low and middle-income countries are premature, and CVDs, diabetes, and cancer are the important killers in this group [8,15,16]. Sub-Saharan Africa faced the greatest challenge from non-communicable diseases besides the communicable diseases among low and middle-income countries [8,17]. A study from Uganda consisting of 320 patients showed that 50% of patients were pre-diabetic and 24% were undiagnosed diabetic [18,19]. Kidney et al. in their study based on 3847 hypertensive patients showed that 10.7% of patients had pre-diabetes and 19.6% had undiagnosed diabetes [20,21]. The age-standardized prevalence of hypertension in Kenya was reported to be similar to the rest of the world at 24.5% but lower compared to the rest of Sub-Saharan Africa at 30% [22].

Another study that analyzed risk factors associated with hypertension and non-communicable disease showed that 75.8% of individuals in the study had four to six risk factors and 10% had seven or more risks [3]. 99.8% of individuals had low fruit intake, 89.5% consumed a high salt diet and 80.3% had insufficient physical activity [3,23]. Other risk factors such as socioeconomic status, marital status, and ethnicity differed between sexes.

Limitations of the study

The key limitations of this study include its retrospective nature and the fact that the study was conducted at a single private practice in one center and as a result, the results may not be generalizable to other centers.

## Conclusions

This study reveals that the use of either the national or alternative guideline-recommended global CVD risk assessment tools was poor and biased against the national guideline-recommended tool with poor documentation of global risk scores. Pharmacological primary CVD prevention with a statin was common practice in hypertensive patients despite the majority of them being in the low-risk CVD category while a high proportion had received specific advice on therapeutic lifestyle interventions.
